# Evidence of West Nile Virus (WNV) Circulation in Wild Birds and WNV RNA Negativity in Mosquitoes of the Danube Delta Biosphere Reserve, Romania, 2016

**DOI:** 10.3390/tropicalmed4030116

**Published:** 2019-08-21

**Authors:** Ana Vasić, Luanda Elena Oșlobanu, Mihai Marinov, Luciana Alexandra Crivei, Ioana Alexandra Rățoi, Adriana Aniță, Dragoș Aniță, Alexandru Doroșencu, Vasile Alexe, Ștefan Răileanu, Predrag Simeunović, Cristian Raileanu, Elena Falcuța, Florian Liviu Prioteasa, Jovan Bojkovski, Ivan Pavlović, Alexander Mathis, Birke Andrea Tews, Gheorghe Savuţa, Eva Veronesi, Cornelia Silaghi, the SCOPES AMSAR training group

**Affiliations:** 1 Institute of Infectology, Friedrich-Loeffler-Institut, Südufer 10, 17943 Insel Riems, Germany; 2 Faculty of Veterinary Medicine, University of Belgrade, Bul. oslobodjenja 18, 11000 Belgrade, Serbia; 3 Faculty of Veterinary Medicine, University of Agricultural Sciences and Veterinary Medicine Iaşi, Aleea Mihail Sadoveanu 3, 700490 Iaşi, Romania; 4 Danube Delta National Institute for Research and Development, Strada Babadag 165, 820112 Tulcea, Romania; 5 Cantacuzino National Medico-Military Institute for Research and Development, Splaiul Independenţei 103, 05096 Bucharest, Romania; 6 Scientific Veterinary Institute of Serbia Belgrade, Vojvode Toze 14, 11000 Belgrade, Serbia; 7 National Centre for Vector Entomology, Institute of Parasitology, Vetsuisse Faculty, University of Zürich, Winterthurerstrasse 266a, 8057 Zürich, Switzerland; 8 Department of Biology, University of Greifswald, Domstrasse 11, 17489 Greifswald, Germany; 9 Faculty for Agriculture, University of Novi Sad, Trg Dositeja Obradovića 8, 21000 Novi Sad, Serbia

**Keywords:** West Nile virus, wild birds, VNT, ELISA, *Aedes* spp., *Anopheles* spp., Danube Delta

## Abstract

West Nile virus (WNV) is a zoonotic flavivirus whose transmission cycle in nature includes wild birds as amplifying hosts and ornithophilic mosquito vectors. Bridge vectors can transmit WNV to mammal species potentially causing West Nile Fever. Wild bird migration is a mode of WNV introduction into new areas. The Danube Delta Biosphere Reserve (DDBR) is a major stopover of wild birds migrating between Europe and Africa. The aim of this study was to investigate the presence of WNV in the DDBR during the 2016 transmission season in wild birds and mosquitoes. Blood from 68 wild birds (nine different species) trapped at four different locations was analyzed by competitive ELISA and Virus Neutralization Test (VNT), revealing positive results in 8/68 (11.8%) of the wild birds by ELISA of which six samples (three from juvenile birds) were confirmed seropositive by VNT. Mosquitoes (*n* = 6523, 5 genera) were trapped with CDC Mini Light traps at two locations and in one location resting mosquitoes were caught. The presence of WNV RNA was tested in 134 pools by reverse transcription quantitative PCR (RT-qPCR). None of the pools was positive for WNV-specific RNA. Based on the obtained results, WNV was circulating in the DDBR during 2016.

## 1. Introduction

West Nile virus (WNV) is a zoonotic flavivirus whose transmission cycle in nature includes wild birds as amplifying hosts and ornithophilic mosquito vectors [1]. Bridge vectors (mosquito species feeding on both birds and mammals) can transmit WNV to other species including humans, horses, and other mammals [2]. Certain birds are the primary reservoir hosts for WNV, but there are variations in their susceptibility to infection based on species [3], reservoir competence, and importance as amplifying hosts. After the first detection of WNV lineage 2 in Europe (2004), it was suggested that long distance migratory birds are responsible for the spread of WNV from Africa [4]. The role of resident (sedentary) and short distance migratory birds in the circulation, maintenance, and spread of WNV was also shown [5].

In Romania, the first severe outbreak occurred in 1996 caused by WNV lineage 1 with 393 confirmed human cases [6], while a WNV lineage 2 outbreak occurred in 2010 for the first time [7]. In the Danube Delta Biosphere Reserve (DDBR) WNV was detected in ticks (*Hyalomma marginatum marginatum* nymph collected in 2013) [8] and mosquitoes (WNV lineage 2 strains in *Culex pipiens, Cx. modestus*, and *Coquillettidia richiardii* mosquitoes in 2015, and in 2016 WNV lineage 2 strains belonging to the monophyletic Central/Southern European group of strains which completely replaced the Volgograd 2007-like strain in the mosquito population) [9]. Mosquitoes of the genus *Culex* are considered to play a major role in the transmission of WNV in Europe [10] but WNV RNA was also detected from pools of *Aedes*, *Anopheles*, and *Culiseta* specimens in previous studies [11,12]. Seroprevalence data for WNV in wild birds in Europe is used for an integrated animal–human-vector approach in WNV surveillance [13]. The aim of this study was to investigate the presence of WNV specific antibodies and RNA in populations of wild birds and mosquitoes, respectively, during the 2016 transmission season in order to assess the circulation of WNV in the DDBR in the frame of a summer training school organized under the capacity-building SCOPES (Scientific cooperation between Eastern Europe and Switzerland) AMSAR project [14].

## 2. Materials and Methods 

### 2.1. Study Area

The DDBR (28.18,05,556 longitude East; 45.450,00,000 latitude North) is a relatively small area (5800 square km) at the end of the Danube’s 2860 km long route through Europe towards the Black sea, protected as a whole by Romania and the UNESCO since 1990. It is famous for its diversity and abundance of birds (362 species) [15], and it is one of the major stopover sites for migratory birds on the way to Africa and back to Europe [16].

**Figure 1:**
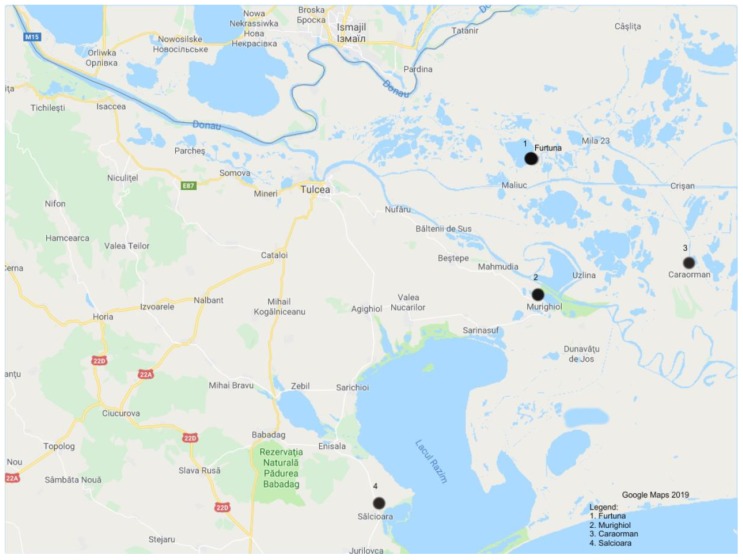
Map of the Danube Delta Biosphere Reserve (DDBR) with sampling locations.

The Sălcioara area is situated in the southern part of the DDBR, in the middle and western part of the Razim-Sinoe lagoonal system. The Furtuna and Stipoc sites are situated in the middle part of the DDBR, in the center of the fluvial delta. The Caraorman site is located also in the middle part of the DDBR, but in the western part of the marine delta. The Murighiol site is in the western part of the DDBR in the southern part of Murighiol village, being the most anthropic sampling point (Figure 1).

### 2.2. Collection and Preparation of Blood Samples from Wild Birds

Collection of blood samples from wild birds was done during three sampling days in summer (19, 21, 23 July 2016) and a second collection point occurred in November 2016 (17 November) in order to track the WNV seroprevalence at the four different locations in the DDBR. Wild birds were caught by placing the catching mist nets (dimensions: 12 m long, 2.5 m high, 5 shelves, 16 × 16 mm) as described in Keyes and Grue, 1982 [17].

Blood was collected from the jugular vein, sera separated and sampled in quantity depending on the size of the bird and stored at −20 °C until further use. Age (adult or juvenile) and gender was identified for almost all birds (Table 1). All caught wild birds were in good condition. After taking blood, the birds were measured, banded, and released. The study was approved by the Scientific Council Ethic Commission of the Danube Delta National Institute for Research and Development. 

### 2.3. Mosquito Collection and Identification 

CDC mini light traps (BioQuip, Rancho Dominguez, CA, USA) with CO_2_ delivered by dry ice as attractant were used for mosquito trapping during two nights (19 and 21 July 2016) at two locations in the DDBR (Furtuna and Caraorman). The traps were placed 1.5 m above ground level in the evening and the trapped mosquitoes were collected in the morning the next day. On the third sampling night (22 July 2016) resting mosquitoes were collected by mouth aspirator (John W. Hock Company, Gainesville, FL, USA) at the Stipoc location. Mosquitoes were collected alive at all three locations, immediately frozen in liquid nitrogen, and identified to genus level according to morphological characteristics given by the identification keys in Becker et al., 2010 [18].

### 2.4. Serological Examination

#### 2.4.1. Competitive ELISA

A competitive ELISA (ID Screen® West Nile Competition Multi-species, IDVet, Grabels, France) was used according to the manufacturer’s instruction to determine the presence of WNV specific antibodies in the collected wild birds’ serum samples [19].

#### 2.4.2. Virus Neutralization Test (VNT)

For confirmation of the positive results obtained by competitive ELISA, a VNT in microtiter format was performed as described in the Manual of Diagnostic Tests and Vaccines for Terrestrial Animals, OIE, 2018 [20]. WNV lineage 1 (Italy, strain 204913/09) was used for the VNT [21]. The results were read after 5 days at 40× magnification. Presence and absence of cytopathogenic effect (CPE) were evaluated for each sample. 

### 2.5. Molecular Examination

#### 2.5.1. RNA Extraction from Mosquito Pools 

Mosquitoes of the same genus were pooled (*n* = 134) and homogenized using a Tissue Lyser II (Qiagen, Hildesheim, Germany), with shaking twice at 30 1/s for 1 min. RNA extractions were performed with the Qiagen RNeasy Mini Kit (Qiagen, Hilden, Germany) according to the manufacturer’s instructions. RNA was eluted in 50 µL of elution buffer and was stored at −80 °C until further use.

#### 2.5.2. RT-qPCR for WNV

In order to determine the presence of WNV specific RNA (target: WNVNS2A) in RNA extracts of mosquito pools, RT-qPCR was performed as described in Eiden et al. 2010 [22]. 

## 3. Results

### Wild Bird Collection and Serological Testing for WNV Antibodies

Altogether 68 wild birds (10 in July and 58 in November 2016) were caught and sampled during this study at four different locations in the Danube Delta Biosphere Reserve (DDBR) (Caraorman *n* = 3, Furtuna *n* = 7, Murighiol *n* = 20, and Sălcioara *n* = 38). Out of the 68 wild birds, 22 were juveniles (hatched within the 2016 season) and 46 were adults. As for the adult birds: 12 were female and 14 male, with gender un-determined for an additional 20 animals as genders were unable to be distinguished based on plumage at the time of sampling. The species composition included migratory (*Acrocephalus scirpaceus*/Common Reed-Warbler, *n* = 3, *Coracias garrulus*/blue roller, *n* = 1), resident (*Cyanistes caeruleus*/eurasian blue Tit, *n* = 1, *Parus major*/great tit, *n* = 1, *Passer domesticus*/house sparrow, *n* = 23, *Passer montanus*/eurasian tree sparrow, *n* = 15, *Picus canus*/grey-headed woodpecker, *n* = 2, *Streptopelia decaocto*/eurasian collared-dove, *n* = 1) and partial migrant species (*Sturnus vulgaris*/common starling, *n* = 21). 

By competitive ELISA, the presence of specific WNV antibodies was shown in 8/68 (11.8%, CI95 11.5%–12.1%) wild birds’ serum samples originating from five species. Four positive samples were collected from juvenile birds (three from the Sălcioara location and one from the Furtuna location) and three were collected from adult birds (one at each location Murighiol, Furtuna, and Sălcioara) whilst in one case of an Eurasian Tree Sparrow (from Sălcioara) it was neither possible to distinguish gender nor age (Table 1). In examined locations, the following positivity rates were registered: 2/7 birds (28.5%, CI95 22.8%–33.9%) from Furtuna, 1/20 (5%, CI95 4.6%–5.4%) from Murighiol, and 5/38 (13.2%, CI95 12.7%–13.7%) from Sălcioara. Confirmatory VNTs were performed on 51 samples collected from two locations (Murighiol and Sălcioara) while 17 samples were not available for further testing due to small quantities of sera. In addition, the insufficient amount of sera did not allow us to carry out neutralization tests with other related flaviviruses circulating in Europe. The VNT results for the ELISA positive samples are given in Table 1. 

A total of 6523 female mosquitoes were collected from three of the four examined locations (Caraorman, Furtuna, and Stipoc) belonging to five genera (Table 2). The mosquitoes were sorted into 134 pools according to genus, with a maximum of 50 individuals each. No specific WNV RNA was found in any of the tested pools.

## 4. Discussion

WNV positive wild birds are considered an important environmental predictor of WNV human risk [23]. Resident birds are responsible for local WNV amplification while infected migratory birds disperse WNV over long distances [24]. Hatch-year birds are thought to have a major role in the amplification of WNV in epizootic transmission [25]. The confirmed seropositivity for WNV registered in this study is 8.8% ± 0.07% (6/68) in wild birds’ sera samples originating from two out of four examined locations (Murighiol and Sălcioara) in the DDBR which is in concordance with findings in neighboring Serbia (8% seropositivity) [26] and Poland (13.3% seropositivity) [27]. The majority of seropositive wild bird species in our study belong to the group of resident birds implying an established transmission cycle of WNV in the DDBR. In contrast, in Germany where WNV was not detected until 2018, antibodies were exclusively found in migratory birds, with the exception of one Hooded Crow *(Corvus corone cornix)* being a short-distance migratory species [28,29]. The geographical distribution of seropositive wild birds of which three were juvenile in two out of four examined locations may indicate the circulation of WNV or other closely related flaviviruses (since the limitations of the performed test exist in relation to cross reactivity) during the 2016 transmission season in the DDBR. In 2016 in the DDBR, 25 out of 227 mosquito pools were tested positive for WNV RNA, all of which were *Cx. pipiens* pools [9]. In the present study, 134 mosquito pools containing 6523 mosquitoes of five genera were all negative for WNV RNA. In a previous study from Romania from 2011 to 2013, WNV RNA was detected in *Cx. pipiens, Cx. modestus, Cq. richiardii, An. hyrcanus, Uranotaenia unguiculata, Ochlerotatus caspius*, and *An. maculipennis* complex [30]. The absence of WNV RNA in the mosquito pools in our study could be due to the short sampling period of three nights in total during July 2016. Even though two ELISA positive grey-headed woodpeckers (*Picus canus*) were sampled in July, the rest of the seropositive wild birds were caught and sampled during November when the probability of WNV positive findings in both wild birds and mosquitoes is higher because of the longer exposure time during the transmission season.

## 5. Conclusions

During summer and autumn 2016, blood sera from six resident and two migratory wild birds of the DDBR had specific WNV antibodies. Overall, a positivity rate of 8.8% was found during this study. The detection of WNV specific antibodies from juvenile resident wild birds indicates infection with WNV or other closely related flaviviruses during the 2016 transmission season. Even though the seropositive wild birds were detected at two different locations (Murighiol and Sălcioara), no WNV specific RNA was detected during this study in mosquito pools in the DDBR.

## Figures and Tables

**Table 1:** Wild birds from Danube Delta Biosphere Reserve that tested positive for West Nile virus (WNV) by competitive ELISA and corresponding Virus Neutralization Test (VNT) results.

Species	Age	Gender	Date of Catch	Location	Competitive ELISA Test	VNT Titer
*Passer montanus/*Eurasian Tree Sparrow	NA	Not distinguishable	21 November 2016	Sălcioara	Positive	1:10
*Passer domesticus/*House Sparrow	Adult	M	21 November 2016	Sălcioara	Positive	1:40
*Passer domesticus/*House Sparrow	Adult	M	17 November 2016	Murighiol	Positive	1:10
*Picus canus/*Grey-headed Woodpecker	Juvenile	F	19 July 2016	Furtuna	Positive	NA
*Picus canus/*Grey-headed Woodpecker	Adult	F	19 July 2016	Furtuna	Positive	NA
*Streptopelia decaocto/*Eurasian Collared-dove	Juvenile	NA	21 July 2016	Sălcioara	Positive	1:20
*Sturnus vulgaris/*Common Starling	Juvenile	NA	21 November 2016	Sălcioara	Positive	1:20
*Sturnus vulgaris/*Common Starling	Juvenile	NA	21 November 2016	Sălcioara	Positive	1:80

NA-not available, M-male, F-female.

**Table 2:** Mosquito genus composition, number of pools, and individual mosquitoes from three different locations within the DDBR.

Mosquito Genus	Location			Total 3 Locations
	Caraorman	Furtuna	Stipoc	
	No. Pools (No. Mosquitoes)	No. Pools (No. Mosquitoes)	No. Pools (No. Mosquitoes)	No. Pools (No. Mosquitoes)
*Aedes*	0 (0)	1 (38)	0 (0)	1 (38)
*Anopheles*	27 (1348)	65 (3181)	5 (250)	97 (4779)
*Coquilletidia*	7 (350)	11 (481)	0 (0)	18 (831)
*Culex*	0 (0)	14 (675)	0 (0)	14 (675)
*Culiseta*.	0 (0)	4 (200)	0 (0)	4 (200)
Total:	34 (1698)	95 (4575)	5 (250)	134 (6523)

Each pool contained up to 50 individual mosquitoes.
